# Light-responsive nanochannels based on the supramolecular host–guest system

**DOI:** 10.3389/fchem.2022.986908

**Published:** 2022-09-21

**Authors:** Jiaxin Quan, Ying Guo, Junkai Ma, Deqing Long, Jingjing Wang, Liling Zhang, Yong Sun, Manivannan Kalavathi Dhinakaran, Haibing Li

**Affiliations:** ^1^ School of Chemical and Environmental Engineering, Hanjiang Normal University, Shi Yan, China; ^2^ Hubei Key Laboratory of Wudang Local Chinese Medicine Research, Department of Chemistry, School of Pharmacy Hubei University of Medicine, Shiyan, China; ^3^ Key Laboratory of Pesticide and Chemical Biology, Ministry of Education, College of Chemistry, Central China Normal University, Wuhan, China

**Keywords:** functional nanochannels, light response, host–guest system, supramolecule, mass transport

## Abstract

The light-responsive nanochannel of rhodopsin gained wider research interest from its crucial roles in light-induced biological functions, such as visual signal transduction and energy conversion, though its poor stability and susceptibility to inactivation *in vitro* have limited its exploration. However, the fabrication of artificial nanochannels with the properties of physical stability, controllable structure, and easy functional modification becomes a biomimetic system to study the stimulus-responsive gating properties. Typically, light-responsive molecules of azobenzene (Azo), retinal, and spiropyran were introduced into nanochannels as photo-switches, which can change the inner surface wettability of nanochannels under the influence of light; this ultimately results in the photoresponsive nature of biomimetic nanochannels. Furthermore, the fine-tuning of their stimulus-responsive properties can be achieved through the introduction of host–guest systems generally combined with a non-covalent bond, and the assembling process is reversible. These host–guest systems have been introduced into the nanochannels to form different functions. Based on the host–guest system of light-responsive reversible interaction, it can not only change the internal surface properties of the nanochannel and control the recognition and transmission behaviors but also realize the controlled release of a specific host or guest molecules in the nanochannel. At present, macrocyclic host molecules have been introduced into nanochannels including pillararenes, cyclodextrin (CD), and metal–organic frameworks (MOFs). They are introduced into the nanochannel through chemical modification or host–guest assemble methods. Based on the changes in the light-responsive structure of azobenzene, spiropyran, retinal, and others with macrocycle host molecules, the surface charge and hydrophilic and hydrophobic properties of the nanochannel were changed to regulate the ionic and molecular transport. In this study, the development of photoresponsive host and guest-assembled nanochannel systems from design to application is reviewed, and the research prospects and problems of this photo-responsive nanochannel membrane are presented.

## Introduction

Ionic and molecular channels play significant roles in most biological activities, such as nerve signal transmission (Kandori et al., 2018), material transfer (Arnspang et al., 2019), and energy conversion (Gouaux and MacKinnon, 2005). Meanwhile, their abnormality may result in various tissue metabolic dysfunctions (Payandeh et al., 2011; Hoon et al., 2014). There arises keen attention over the research on biological protein channels. However, biological protein channels are unstable and easily become inactive *in vitro* conditions, and these factors highly limit the study of their relevant biological behaviors (Xie M. Q. et al., 2018). To explore and gain more information on similar biological processes, biomimetic nanochannels were proposed as the synthetic counterparts of biological channels, which can mimic their functions, with the advantage of excellent mechanical properties and good chemical stability than biological channels (Guo et al., 2013) *in vitro* conditions. Scientists have successfully built a variety of stimulus-responsive biomimetic nanochannels, such as light (Ali et al., 2012), pH (Zhang et al., 2016), temperature (Wang R. et al., 2017), electric potential (Liu et al., 2016), magnetic (Chang et al., 2020), and others (Cao et al., 2021). Comparatively, light stimulation has benefits of non-invasiveness, accuracy, easy operation, and repeatable stimulus-response (Cheng et al., 2022) than others. Moreover, light stimulation can be applied at a selective location with control on intensity and illumination time; this can induce conformational changes (Xiao et al., 2016a). Artificial ionic or molecular gates are constructed by introducing a host–guest system into the nanochannels, unifying good reversibility and high selectivity (Li et al., 2022; Zhang et al., 2022).

**SCHEME 1:**
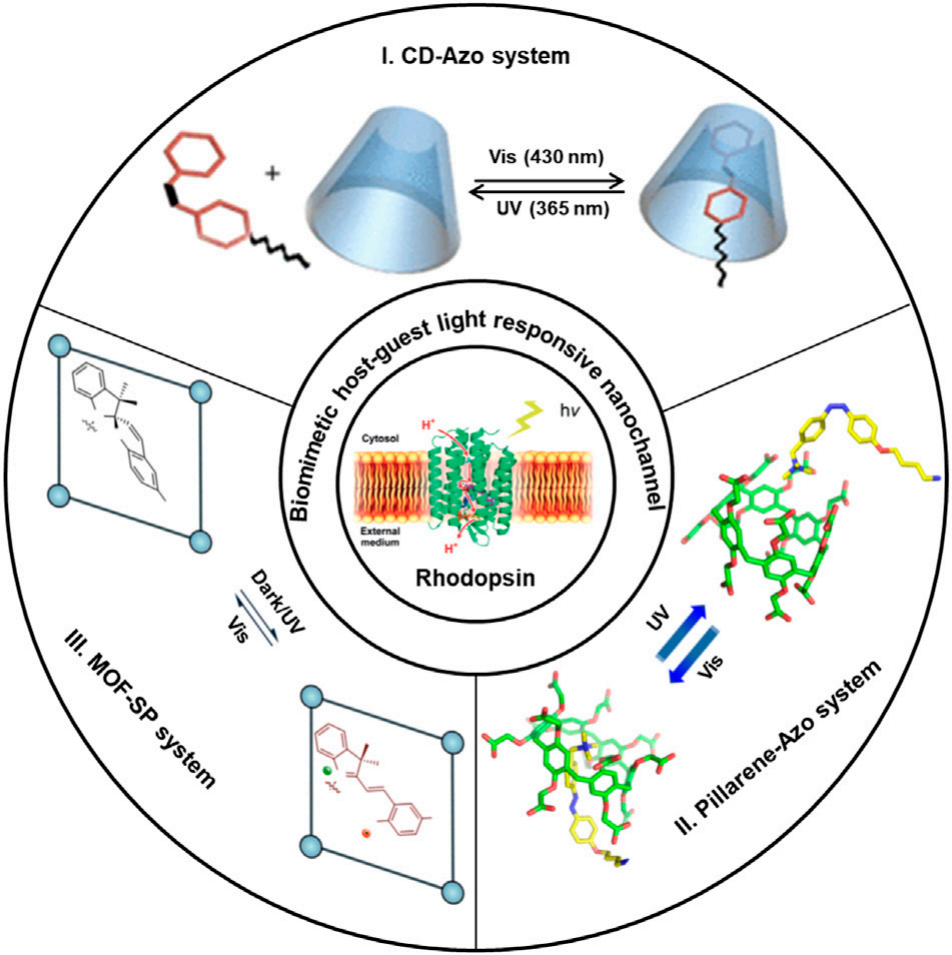
Different host–guest system-assembled nanochannels.

In nature, light-responsive protein nanochannels are also important parts of organisms (Shichida & Matsuyama, 2009). For example, rhodopsin as a light-responsive protein-membrane channel consists of opsin and retinal (Rinaldi et al., 2014). The natural chromophore of retinal has light-responsive properties and plays crucial parts in visual signal transduction and energy conversion (Fujita et al., 2020). Its abnormalities will lead to tissue dysfunction of blocked visual signaling transmission; this eventually results in diseases of floaters and blindness (DePoli et al., 2016). Inspired by light-responsive protein channels in cell membranes, scientists have fabricated light-responsive channels with photoswitches of molecules, such as azobenzene (Li et al., 2016), spiropyran (Terpstra et al., 2017), and retinal (Postnikova et al., 2019) to realize the light-controlled behaviors. Based on the structural change of photosensitive molecules, light-responsive biomimetic nanochannels were constructed to realize the regulation of ionic and molecular transport behaviors (Zhang et al., 2015; Chun et al., 2018). The combined effect of stimulus responsiveness and host–guest interaction together brings the benefit of selective and sensitive transport or recognition of ions and molecules across the biomimetic nanochannels. Moreover, light responsiveness and host–guest interactions are reversible. Light-responsive host–guest systems have the characteristics of selective recognition, non-covalent bond interaction, and reversible assemble behaviors (Petermayer & Dube, 2018; Samanta et al., 2018; Liu et al., 2021). Based on the light-responsive host–guest system of reversible interaction, light can not only change the inner surface properties of nanochannels (Zhang Q. Q. et al., 2012) to realize the regulation of selective identification and transmission behaviors but also can achieve the specific molecular controllable release of host or guest molecules (Angelos et al., 2009). At present, nanochannels were functionalized with a variety of molecular scaffolds, including macrocyclic hosts such as pillar[n]arene (Li et al., 2022), cyclodextrin (CD) (Su et al., 2022), and metal–organic skeleton (MOF) (Zheng et al., 2022), either through covalent bonding modification or host–guest assemble methods (Scheme 1). Photo-responsiveness of azobenzene, spirogyra, and retinal results from their geometrical or chemical transformation attained by them with the exposure of light; this ultimately results in complexation and decomplexation of the host–guest leading to changes in the hydrophobicity of nanochannels. In final, this series of actions can regulate the selective transport of ions and molecules by light.

## Photoresponsive host–guest system-assembled nanochannels

### Cyclodextrin–azobenzene photoresponsive host–guest system-modified nanochannels

Photoresponsive hydrophobicity of nanochannels can switch rapidly and repeatedly between different states; this brings extensive attention. However, regulating the transport of substances in hydrophobic nanochannels controlled by light and electric fields is an interesting and challenging task. The cumulative effort of hydrophobic and hydrophilic interaction was needed for the enrichment and transport of ions and molecules. Cyclodextrin (CD) as an interesting macrocyclic scaffold possesses intracavitary hydrophobic and extracavitary hydrophilic cyclic oligosaccharides consisting of 6, 7, or 8 D-glucose units linked through α-1,4-glycosidic bonds assigned as α-, β-, and γ-CD, respectively. It plays important roles in chiral recognition, molecular switching, fluorescence imaging, supramolecular self-assembly, controlled drug release, and enzyme simulation. β-CD with a suitable size of the cavity has unique advantages in industrial applications, though its host–guest complexation with a larger number of small organic molecules and thus brings changes in their physical and chemical properties.

**FIGURE 1:**
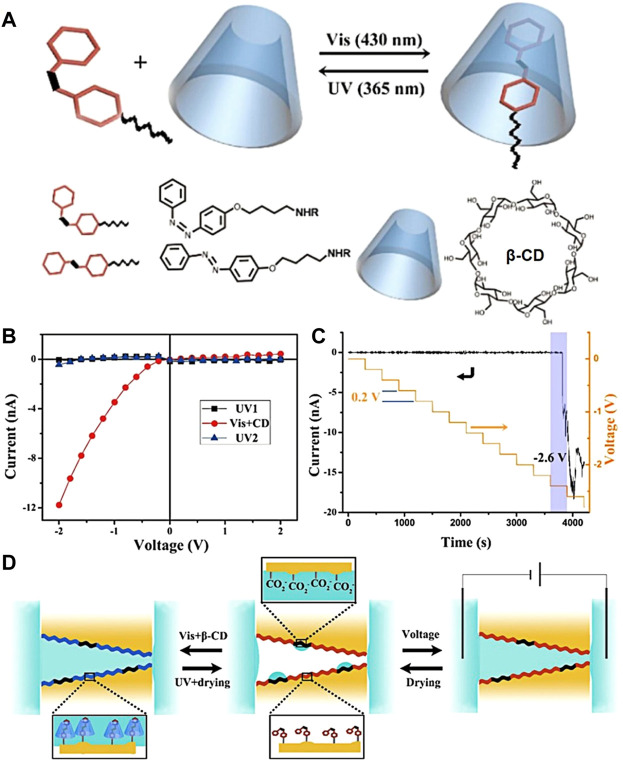
**(A)** Schematic diagram of optical inclusion and de-inclusion of azobenzene (Azo) derivatives and β-CD; **(B)** changes in ionic current in Azo modified nanochannels before and after β-CD assembly under UV and visible light; **(C)** hydrophobic nanochannels modified by Azo opened under −2.6 V; **(D)** schematic diagram of the assembly of β-CD-Azo host-guest compounds in nanochannels regulated by photo and electricity (G. H. Xie, P. Li, Z. J. Zhao, Z. P. Zhu, et al., 2018) Copyright @ 2018 (ACS).

Initially, the light-controlled host–guest system of azobenzene with cyclodextrin was reported. This light-responsive system works based on the *cis*-*trans* isomerization of the azobenzene unit (Figure 1A); here, β-CD forms a stable complex with the *cis*-isomer of azobenzene, and light-induced photo-isomerization into the *trans* form resulted in decomplexation (Suzuki et al., 2012; Harada et al., 2014; Kamiya & Asanuma, 2014). Such a dynamic light-responsive host–guest interaction was well utilized by Lei Jiang’s group to fabricate light-responsive azobenzene-functionalized nanochannels, which can establish light-responsive reversible host–guest interactions with β-CD. Hereby, the complexation and decomplexation between β-CD and azobenzene derivatives highly alter the hydrophobicity of the inner surface of nanochannels, which results in controlled molecular or ionic transport across the membrane (G. H. Xie, P. Li, et al., 2018). Initially, to illustrate the photo-responsive host–guest interaction between β-CD and Azo in nanochannels, the host–guest interaction of β-CD and Azo in solution was correspondingly explored through UV-Vis titration, in which the enhancement in the absorption intensity evidences the formation of an inclusion complex. Under UV light irradiation, Azo undergoes a geometrical transformation from *trans* to *cis*. Thus, the absorption of β-CD and *cis*-Azo is almost the same as the absorption of pure *cis*-Azo; this confirms that *cis*-Azo hardly interacts with β-CD (Figure 1B). A similar trend was also observed within the nanochannel when β-CD was introduced into the *trans*-Azo-functionalized nanochannel through host–guest assembly under visible light. Its wettability changes due to the hydrophilic nature of the outer surface of CD. Upon irradiation with UV light, Azo undergoes photo-isomerization of *trans* to *cis*. This results in decomplexation, eventually leading to increased hydrophobicity. It finally affects the transport behavior of ions and molecules in an aqueous solution across the membrane (Xiao et al., 2016b). In addition, light and electric dual-responsive molecular nanochannels were also reported by Wang Y. et al. (2017). On reaching an applied potential of -2.6 V, nanochannels attain an “*on”* state and facilitate molecular or ionic transport, while it closes with a potential lower than -2.6 V (Figure 1C). Here, molecular/ionic transport can also be regulated by light. Light and electric regulated-“*on”* and “*off”* states of nanochannels will have a good applicative prospect in optical and electronic logic gating (Figure 1D).

**FIGURE 2:**
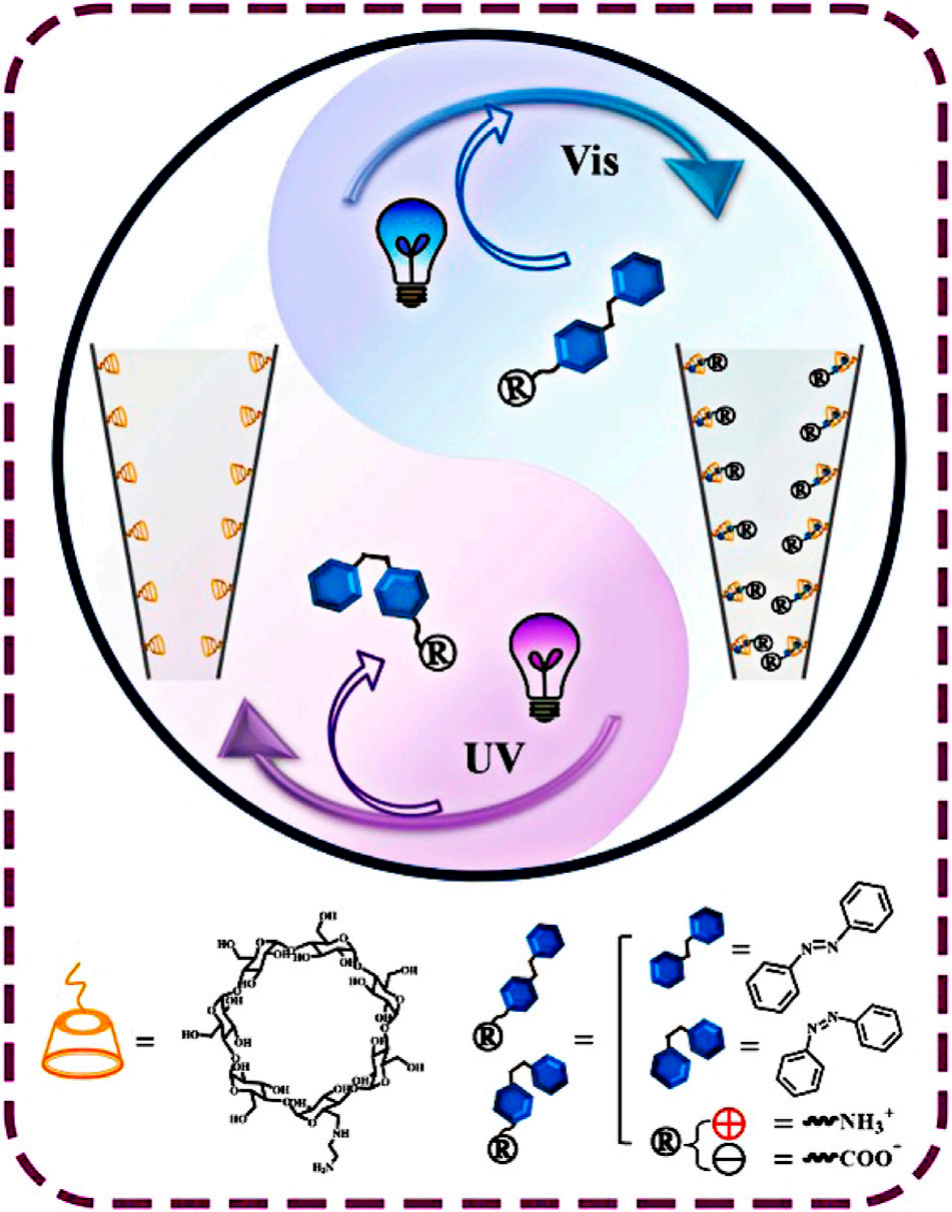
Schematic diagram of reversible assembly of azobenzene derivatives in NH_2_-β-CD-modified nanochannels (Liu et al., 2018) Copyright @ 2018 (Springer Nature).

Moreover, Liping Wen’s group reported a nanofluid diode with replaceable functional groups on polyimide conical nanochannels by the inspiration of biological processes (Liu et al., 2018). Here, under visible light irradiation, the azophenyl group with amine and carboxylic acid groups exists in *trans*-conformation and is bound to β-CD; this results in pH-controlled positive and negative charging on the inner surface of nanochannels. Furthermore, with the exposure to UV light, it gets de-capsulated from the inner surface of nanochannels upon photo-isomerization of the Azo group from *trans* to *cis* conformation, which corresponds to the ionic current in the nanochannel returned to the initial value. Therefore, the pR (positive rectification degree, pR = log [|I_-2 V_/I_+2 V_|]) value switched between -0.7 and 0.1 for Azo-NH_2_, and between 0.85 and -0.15 for Azo-COOH with switching in pH. Moreover, this was recyclable over a period of five times (Figure 2). This study provides a new method for constructing nanofluidic platforms with replaceable surface functional groups and has broad application prospects in the fields of photosensitive nanofluidic devices (Sparreboom et al., 2009), drug transport and release, and nanofluidic logic gates (Guliyev et al., 2011).

**FIGURE 3:**
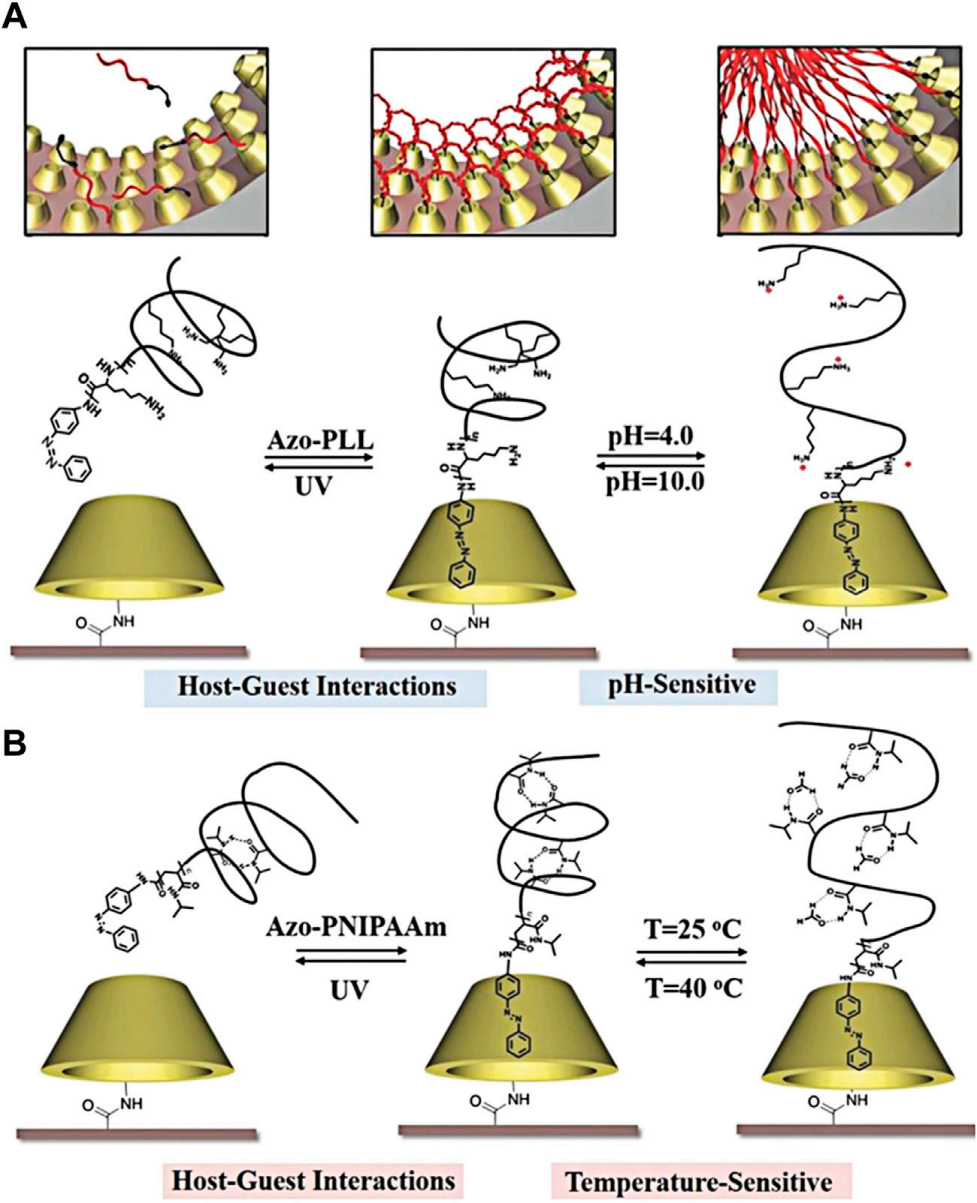
**(A)** Schematic diagram for the reversible assembly of poly-L-lysine-derived azobenzenes assembled in α-CD-modified nanochannels through the host–guest interaction with light- and pH-responsive properties; **(B)** schematic diagram for the reversible assembly of poly-n-isopropyl acrylamide-derived azobenzenes assembled in α-CD-modified nanochannels through the host–guest interaction with light- and temperature-responsive properties (Liu Y. Z. et al., 2017) Copyright @ 2017 (Wiley).

In addition, Fan Xia’s group reported the host–guest system of azobenzene functionalized poly-L-lysine (PLL) and poly-n-isopropylacrylamide (PNIPAAm) azobenzene over the 3-amino-3-deoxy-α-cyclodextrin (α-CD)-crafted artificial nanochannel. In this functional nanochannel, pH responsiveness was attributed to the presence of PLL (Figure 3A). Meanwhile, PNIPAAm brings the temperature response (Figure 3B) (Liu N. N. et al., 2017). Due to weak intermolecular host–guest interactions, the molecules of PLL and PNIPAAm coexist on the inner surface of the nanochannel. Interestingly, PLL and PNIPAAm functional nanochannels have six responsive states to three environmental stimuli, namely, light, pH, and temperature through reversible host–guest assembly and disassembly of PLL and PNIPAAm over four cycles (Pace et al., 2007). Moreover, α-CD fabricated nanochannels possess the benefit of accommodating PLL, PNIPAAm individually, and the sequence of PLL + PNIPAAm. Both the derivatives of azobenzene molecules can be peeled off the α-CD-modified nanochannels under the irradiation of UV light. These systems are classified into dual-responsive coexistence and “plug-and-play” templates (Liu et al., 2013), which have individually or continuously reversible properties. A cultivation of strategy exploits weak interaction to simultaneously, transiently, and reversibly modify multiple responses in given artificial nanochannels.

**FIGURE 4:**
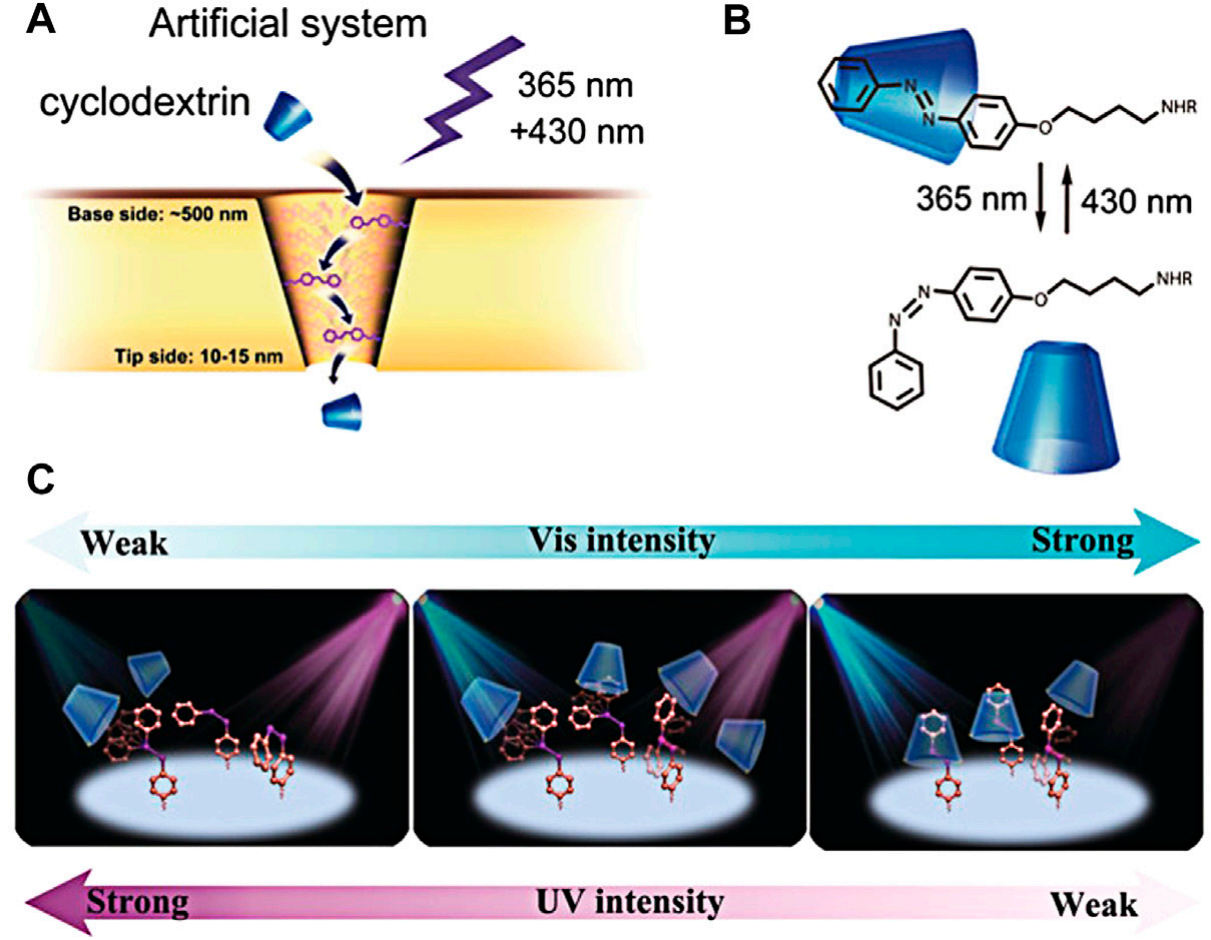
**(A)** Cyclodextrin transporter of artificial nanochannel systems; **(B)** schematic diagram of inclusion and de-inclusion of cyclodextrin–azobenzene in UV/visible light response; **(C)** selective transport behavior of azo groups on the inner surface of nanochannel under the different irradiation of visible light (blue) and ultraviolet light (purple). A single high intensity of visible or ultraviolet light will reduce the transmission rate of β-CD. Only the matching intensity of visible and ultraviolet light can promote the transmission of β-CD (Xie et al., 2018b) Copyright @ 2018 (Wiley).

Based on these studies, Lei Jiang’s group reported a light-driven artificial molecular motor for the transport of β-cyclodextrin (β-CD) by utilizing its host–guest interaction with the azobenzene molecule (Figure 4A) (Xie et al., 2018a). This transport system was constructed by the fabrication of nanochannels with an azobenzene unit, over which β-CD was bounded by host–guest interaction. However, the cooperative irradiation of UV and visible light on selective regions can successfully balance the *cis-trans* isomers of azobenzene; this leads to a balance of complexation and de-complexation of azobenzene with β-CD, and eventually results in the efficient transport of β-CD over the membrane (Figure 4B) (Liu et al., 2018).

Instead, their results suggest that the intensity of visible and ultraviolet light must match to efficiently transport β-CDs through azobenzene-modified nanochannel membranes based on the host–guest molecular motor system. If the irradiative intensity of UV and visible light is not balanced, the sequence of *cis* and *trans*-isomers of azobenzene will be random, which means that the artificial molecular motors cannot capture/release β-CD (Figure 4C). Photo-responsive reversible host–guest interaction in the confined space of nanochannels was well utilized to construct the artificial molecular motor to transport molecules across nanochannel membranes. During these transport processes, the reversible photo-isomerization for *cis*-*trans* isomerism of azophenyl groups enables it to act as a filter, stirrer, and transporter to selectively move β-CD molecules through nanochannel membranes. But this system cannot transport β-CDs along a reverse concentration gradient as a light-driven ionic pump. However, this approach provides a viable path for building rotating motors capable of moving cargo over long distances through a continuous capture and release process (Fujiwara & Imura, 2015). By functionalizing these Azo derivatives with other molecules, such as DNA aptamers or molecular cages can specifically for host–guest interactions with β-CDs molecules fabricated on the nanochannels, the proper tuning of complexation and decomplexation by balanced irradiation of UV and visible light facilitates the selective transport of these biomolecules across membranes. These kinds of systems have potential applications in drug delivery and separation. If this process is guided by chemical gradients and electric or magnetic fields, it can also be seen as a precursor to artificially driven molecular pumps. In addition, this artificial photodynamic molecular motor can lead to a better understanding of similar biological tissue processes.

**FIGURE 5:**
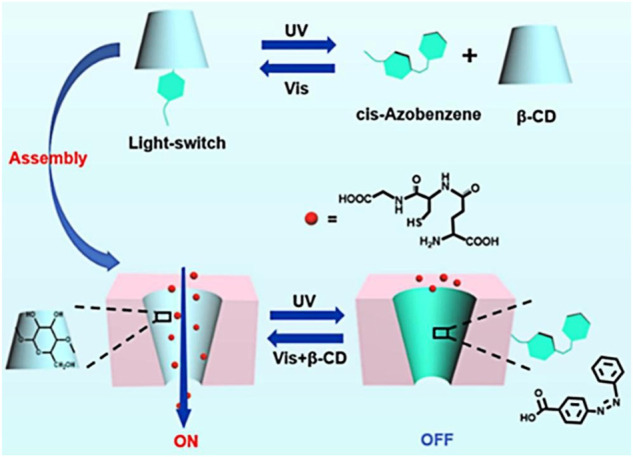
β-CD-carboxyl azobenzene modified assembly into nanochannels in UV/visible light response regulating glutathione transport (Wang et al., 2021) Copyright @ 2021 (Elsevier).

Due to the important role of glutathione (GSH) in living organisms (Lin et al., 2018), the regulation of its transmembrane transport behaviors has attracted wide interest (Jin et al., 2016). For example, our group has modified the photo response of carboxy derivative azobenzene into a nanochannel and further assembled β-CD into this functional nanochannel (Wang et al., 2021). This light-controlled host–guest system provides an “*on”* and “*off”* state for glutathione transport under visible and UV light, respectively. The “*off”* state of glutathione transport attributes to the decomplexation of the host–guest system, which ultimately results from photo-isomerization of the Azo unit from the ‘*trans’* configuration into the *‘cis’* isomer (Figure 5). Such a study on the transport behavior of critically important biomolecules *in vitro* conditions will be much supportive as a synthetic model for exploring the transport behavior of these molecules under *in vivo* conditions.

### Host–guest-assembled light-responsive nanochannel systems with pillararene

In 2008, pillar[*n*]arene was first reported as a new macrocyclic host molecule (Ogoshi et al., 2008). According to the number of benzene units forming pillar[*n*]arene, it can be divided into pillar[*n*]arene (n = 5, 6, 7, 8, 9, 10). It has been widely studied as a macrocyclic host molecule due to its easy synthesis, good structural symmetry, multifunctional sites, easy derivatization and functionalization, strong recognition ability, and planar chirality in recent years (Ogoshi et al., 2016). Chemists have successfully constructed a variety of supramolecular functional materials with novel structures using pillararene and applied it in various fields, such as the controlled release of drugs, biomedicine, stimulation-responsive materials, and self-healing materials (Yang et al., 2015a; 2015b; Qu et al., 2015; Xue et al., 2015). Among them, Feihe Huang’s group assembled ethoxy pillar[6]arene and quaternary ammonium salt of azobenzene together to construct a host–guest complex in an aqueous solution, showing a good UV/visible light response to host–guest recognition performance (Yu et al., 2012).

**FIGURE 6:**
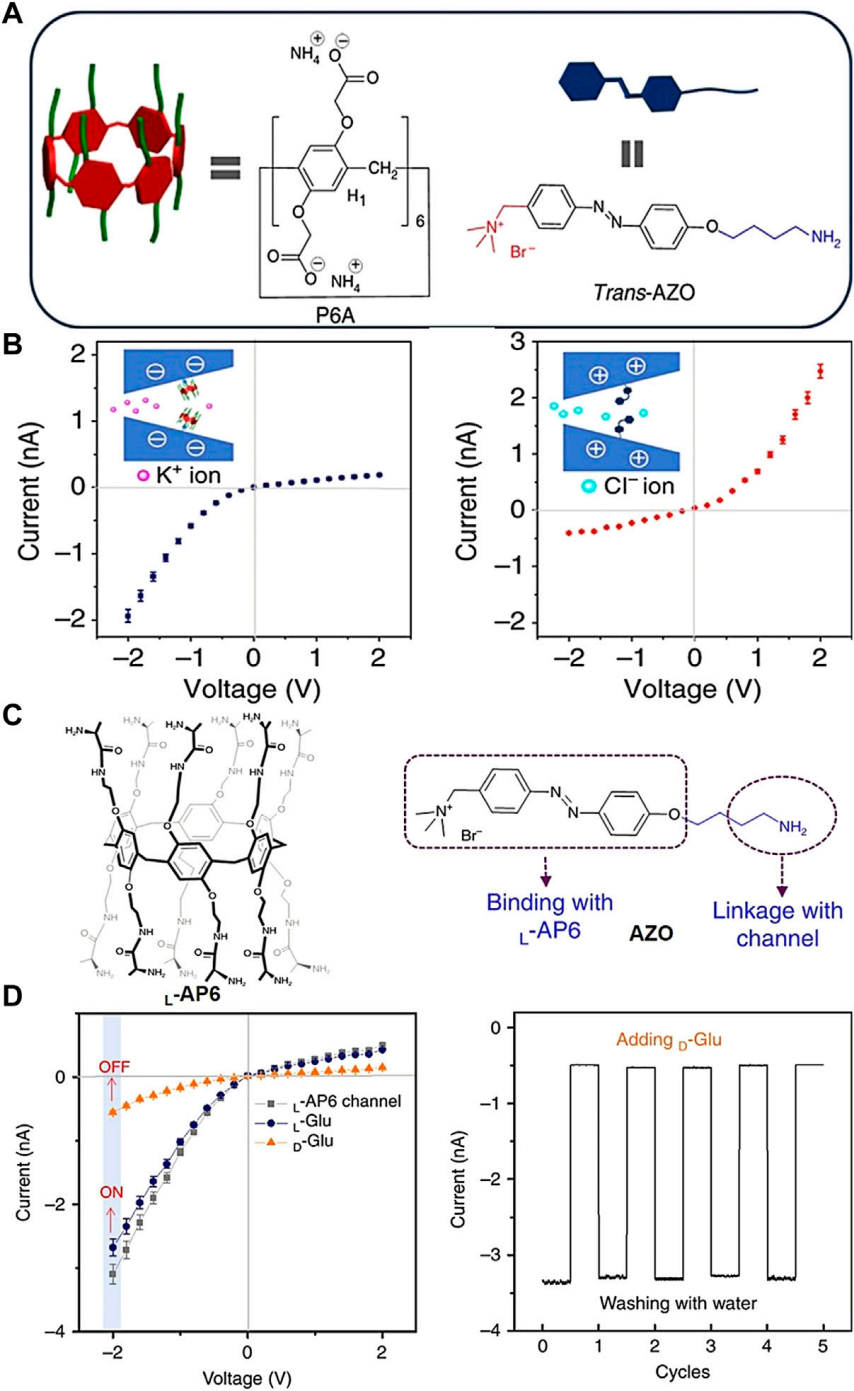
**(A)** Molecular structure diagram of carboxylate-derived pillar[6]arene and quaternary ammonium azobenzene, Copyright @ 2017 (Springer Nature); **(B)** under visible light irradiation, carboxylate-derived pillar[6]arene-assembled nanochannels showed selective transport behaviors for cations, Copyright @ 2017 (Springer Nature). After irradiated by UV light, quaternary ammonium azobenzene-modified nanochannels showed selective transport behaviors for anions (Sun et al., 2017); **(C)** molecular structures of chiral alanine pillar[6]arene and quaternary ammonium azobenzene, Copyright @ 2018 (Springer Nature); **(D)** D-glucose responds to current changes in ionic-gated nanochannels (Sun et al., 2018) Copyright @ 2018 (Springer Nature).

Based on the established UV/visible light-controlled host–guest complexation of pillar[*n*]arene, we reported the UV/visible light-responsive wrapping and unwrapping performance of negatively charged carboxylate salts of pillar[6]arene with azobenzene molecules in solution (Figure 6A). Then, this pillar[6]arene was assembled into the positively charged quaternary ammonium salt of azobenzene-modified nanochannel (Sun et al., 2017). The surface charge of nanochannels was changed to achieve selective transport behaviors for different ions on account of the UV/visible light-responsive binding properties between pillar[6]arene and azobenzene (Figure 6B). Under visible light irradiation, the nanochannels were assembled by carboxylate-pillar[6]arene, showing the selective transport of cations (K^+^). After irradiation by UV light, pillar[6]arene fell off and the positive charge of the quaternary ammonium on azobenzene was exposed, which was conducive to the selective transport of anions (Cl^−^) (Vlassiouk et al., 2009). Based on the regulation of UV/visible light, pillar[6]arene and azobenzene-assembled nanochannels further achieved the regulation of ATP transmembrane transport behaviors. In addition, chiral alanine pillar[6]arene was designed, synthesized, and assembled into quaternary ammonium azobenzene-modified nanochannels (Figure 6C). Based on the chiral selective interaction between L-alanine-derived pillar[6]arene and D/L-glucose, a biomimetic ionic gated response of glucose enantiomers was constructed (Sun et al., 2018). These chiral nanochannels can be “closed” by D-glucose and “opened” by L-glucose, which shows good reversibility to glucose enantiomers (Figure 6D). This system not only contributes to our understanding of the biological and pathological processes caused by the gating of glucose-sensitive ionic transport but can also be applied to biological processes such as selective delivery and controlled release of drug molecules.

**FIGURE 7:**
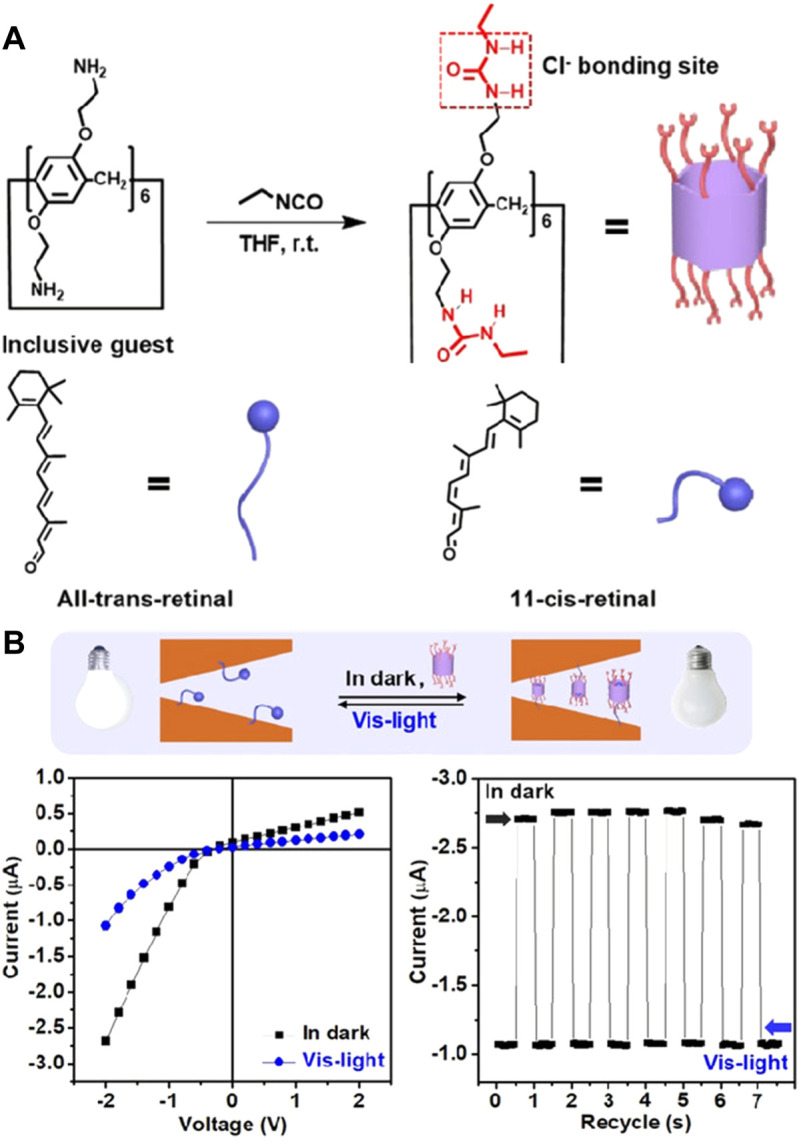
**(A)** Synthesis process of the ethyl urea-derivative pillar[6]arene and the cis-trans structure change of the retinal molecule; **(B)** changes in the ionic current in the nanochannel before and after irradiation by visible light. This process was recycled seven times by the test of I-T current (Quan et al., 2021) Copyright @ 2021 (Wiley).

As a widely distributed energy, visible light has the advantages of no pollution and no harm, and is widely applied in various stimulus-responsive functional materials (Xu & Wang, 2022). Moreover, the natural light-responsive ionic nanochannels of rhodopsin are regulated by visible light and realized the conduction of visual nerve signals (Mous et al., 2022). Therefore, the construction of visible light-responsive ionic transport nanochannels is conducive to deepening the understanding of similar biological processes. It was found that rhodopsin is composed of opsin and retinal. This protein channel is opened and closed by the structural changes of retinal to realize the regulation of ionic transport behaviors, which is the visible light-response of the natural chromophore (Broser, 2022). Inspired by natural rhodopsin, different light-responsive nanochannels have been constructed by the introduction of light-sensitive molecules (Zhang M. H. et al., 2012; Xu et al., 2016). However, it is a challenging task to regulate the selective transport of specific chlorine ions by visible light. To overcome this challenge, the natural chromophore of retinal has been applied to construct visible light-responsive nanochannels (Quan et al., 2021). Furthermore, the molecules with urea structures can realize the selective response of chloride ions by hydrogen bonding interactions (Capici et al., 2011). Pillararene, as a novel generation of macrocyclic supramolecular compounds, is widely applied in the recognition of ions and molecules by the host–guest interaction (Jie et al., 2018). Its side chains with more active sites provide a further derivative functional structure, and the hydrophobic cavity can realize the inclusion of small organic molecules to form the host–guest complex (Li et al., 2019). Therefore, the ethyl urea-derivative pillar [6]arene was designed and synthesized as chloride ionic receptors by a one-step reaction with isocyanate (Figure 7A). Based on the visible light response of retinal molecules, the inclusion and liberation of host–guest complexes were realized. The selective transport of chlorine ions was regulated by visible light in the nanochannels (Figure 7B). This work provides the chemical model for us to deepen our understanding of similar physiological processes. It also provides a potential application for a visible light control release system and energy conversion devices.

**FIGURE 8:**
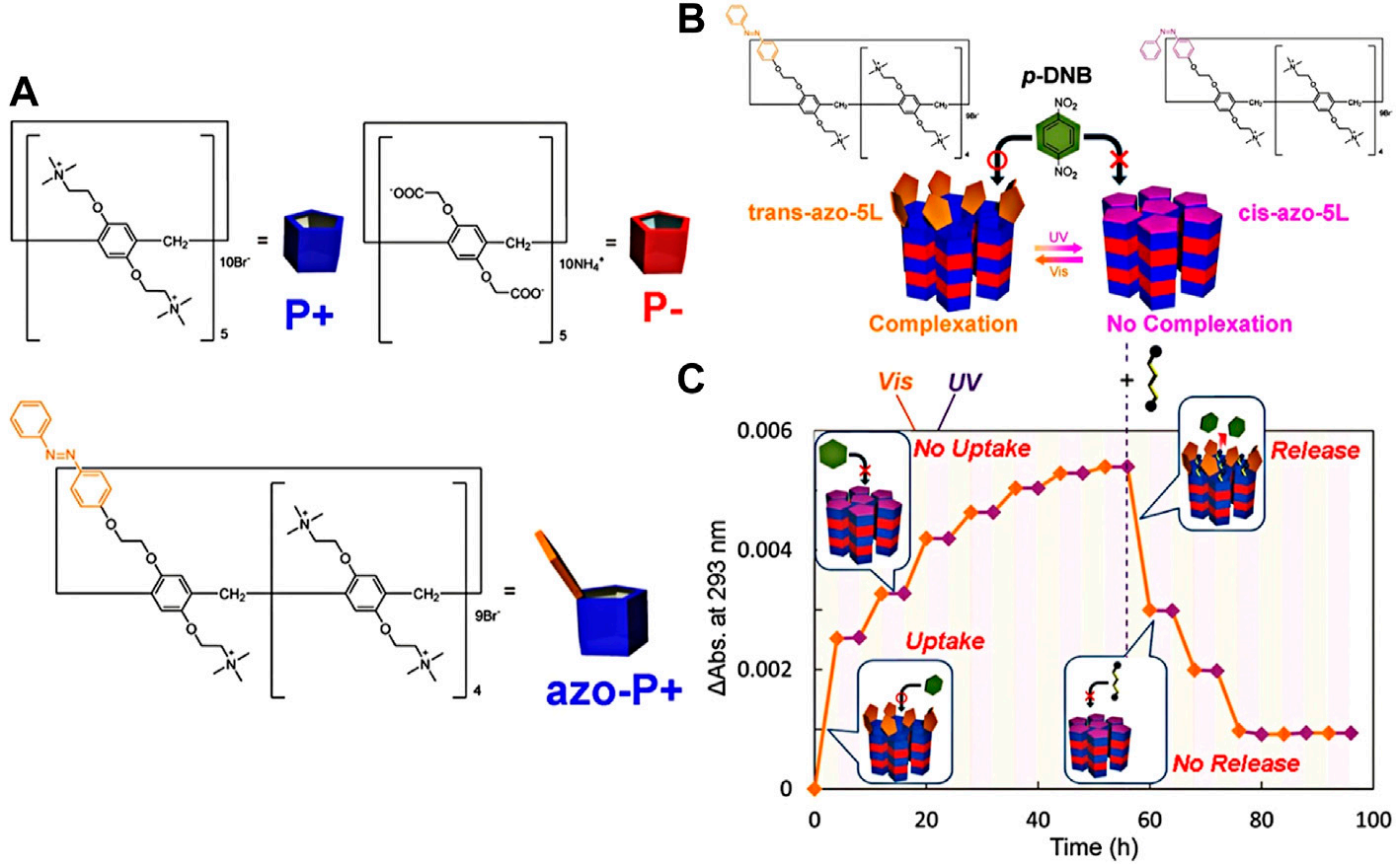
**(A)** Chemical structures of cationic (P+) and anionic (P−) pillar[5]arene, and azobenzene-derived cationic pillar[5]arene (AZo-P+); **(B)** schematic diagram of photo-responsive p-DNB adsorption in sub-nanometer molecular channels; **(C)** absorption, storage, and release of the guest molecule of p-DNB are regulated by UV and visible light and the addition of excessive 1,4-dicyanbutane in the solution (Ogoshi et al., 2018) Copyright @ 2018 (ACS).

Pillararene with the sub-nanometer of the macrocyclic cavity is similar to the size of a biological protein channel (Han et al., 2010). Therefore, Tomoki Ogoshi’s group assembled cationic (P+) and anionic (P-) pillar[5]arenes on a solid surface through a layer-by-layer electrostatic interaction (Figure 8A). Azobenzene-derived cationic pillar[5]arene (azo-P+) was assembled on the top layer to construct the sub-nanometer molecular channel with photo-responsive azo groups (Ogoshi et al., 2018). Thus, a new generation of pillararene molecular channels with sub-nanopores was prepared at a molecular level with light-responsive properties. Based on the UV/visible light-responsive cis-trans isomerization of azo groups, sub-nanometer molecular channels can regulate the absorption, storage, and release of p-dinitrobenzene (p-DNB). It is pointed out that the azo-derived pillar[5]arene did not interfere with the absorption and release of p-DNB in trans isomerization under visible light. When azo was converted into a cis-structure under UV light, the subnanometer molecular channel of pillar[5]arene was blocked in the absorption and release of p-DNB (Figure 8B).

To further demonstrate the light-responsive performance of the sub-nanometer molecular channel, it was assembled with trans-azobenzene cationic pillar[5]arene and adsorbed with p-DNB, immersed in an excessive competitive guest molecule of 1,4-dicyanbutane solution, which successfully induces the release of p-DNB from molecular channels. After UV light irradiation, the trans-azo conversed to a cis-structure. Even in the solution of 1,4-dicyanbutane, there is no release of p-DNB. Based on the use of azo as the photo-responsive molecules, photo-responsive reversible absorption, storage, and release of p-DNB have been demonstrated in the pillararene molecular channel (Figure 8C). This study provides a novel and effective method for the construction of multifunctional sub-nanometer molecular channels to regulate the reversible process of absorption, storage, and release of neutral small molecules.

### Host and guest-assembled light-responsive nanochannels with MOFs

**FIGURE 9:**
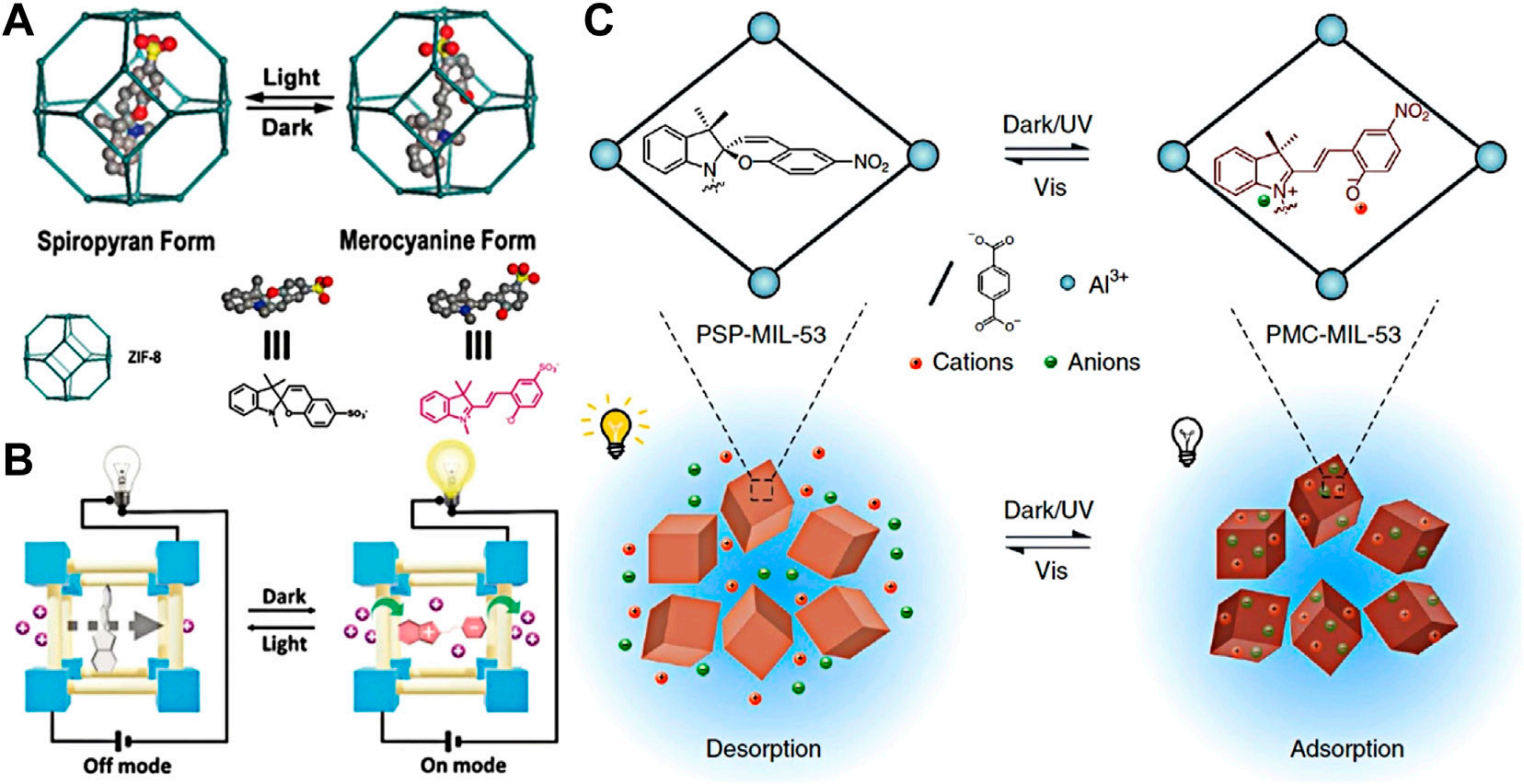
**(A)** Photoisomerization of SP and MC forms in ZIF-8, Copyright @ 2020 (Wiley); **(B)** photoisomerization of SP in the ZIF-8 cavity leads to the on-off state for proton transport (Liang et al., 2020) Copyright @ 2020 (Wiley); **(C)** adsorption and desorption of ions regulated by the UV/visible light response of spiropyran in the MIL-53 MOF (Ou et al., 2020) Copyright @ 2020 (Springer Nature).

Metal–organic frameworks (MOFs) are the kind of porous crystalline materials with large specific surface area, diversified skeleton composition, adjustable pore chemistry, and channel structure (Furukawa et al., 2013). These attractive advantages make MOFs promising not only as substrates of multifunctional composites (Stock & Biswas, 2012; Li et al., 2018) but also as platforms for molecular and proton transport nanochannels (Shimizu et al., 2013). In recent years, the application of MOFs in proton conduction has been widely studied, and a high proton conductivity has been obtained (Ye et al., 2017; Guo et al., 2018). At present, the studies of proton-conductive MOFs mainly focus on enhancing proton conductivity for potential application in fuel cells, while the photo-regulated proton conduction in MOFs is rare. However, a natural proton pump can be driven by light for signal transduction and energy conversion. Recently, Banglin Chen’s group simulated a biological proton pump to achieve proton transfer in response to external stimuli of light. Using photosensitive molecules of spiropyran (SSP) as molecular switches, SSP@ZIF-8 hybrid films were prepared by solid-constrained transformation at room temperature using zinc hydroxide nanostrands (ZHNs) and SSP composite films (Liang et al., 2020). Spiropyran as photosensitive molecules has different conformations irradiated by UV/vis light. For example, Smirnov’s group constructed a light-induced switch to control the wettability of the nanochannel by the modification of photochromic spiropyran (Vlassiouk et al., 2006). Therefore, a ZIF-8 layer was formed on the surface of SSP/ZHN films by immersing in 2-methylimidazole (Hmim) mixed solution. It prevented SSP from escaping from SSP/ZHNs films into the solution and made SSP *in situ* encapsulated into a ZIF-8 crystal (Liu N. N. et al., 2017). The structure of SSP can be reversibly switched in response to photo stimuli by encapsulating the photosensitive molecules of spiropyran sulfonate into the ZIF-8 crystal (Figure 9A).

The structure of SSP can produce different flowing acidic protons, which has high photo switched proton conductivity in the MOF mixed films. Thus, the light regulation of proton conduction in MOF-assembled film was realized. The sulfonate and phenol groups in SSP with the form of merocyanine (MC) significantly improve the proton conductivity of SSP@ZIF-8 films in the dark by forming a hydrogen bond network between the water molecules and these hydrophilic groups in the MOF cavity (Yang et al., 2017). The advantages of high isomerized yield, fast conversion rate, and great difference in hydrophilicity and hydrophobicity between the MC and spiropyran (SP) form of water-soluble molecules made the MOF-SSP films with excellent photo-responsive switching performance. The SSP@ZIF-8–10% film not only had a high proton conductivity of 0.05 S cm^−1^, but also achieved a fast photo response within five cycles and an extremely high *on/off* ratio (2.8×10^4^) under visible light irradiation at 75°C and 95% relative humidity (RH). To expand the practical application of SSP@ZIF-8 films, the first optical switch of proton-conductive films was assembled into the light control devices for remote control of LED lighting (Figure 9B). This work provides the possibility for the application of switched proton-conductive materials in optical switching devices (such as proton-conductive field effect transistors), and also provides a platform for the manufacture of novel intelligent proton solid films.

Biological systems are adept at using sunlight in a range of life processes, including photosynthesis (Barber, 2009), photocatalysis (Zhu et al., 2018), and cell excitation and inhibition (Govorunova et al., 2015). Furthermore, sunlight is the most abundant renewable energy on Earth (Blankenship et al., 2011). The development of a desalination adsorbent using sunlight for regeneration is expected to provide an energy-saving and environmentally sustainable solution for desalinating seawater (Tao et al., 2018). In particular, the photoisomers of spiropyran (SP) can be excited to merocyanine (MC) under dark or UV irradiation to achieve structural isomerization (Klajn, 2014). The positively charged indole group and negatively charged phenolic acid group on MC can act as anion and cation binding sites, respectively, to adsorb salt from brine. In addition, the isomer of MC can be accelerated back to SP by visible light (vis). Based on this photoisomerization of SP, we also assumed that the cations and anions adsorbed on MC can be released in the process of light-driven isomerization of SP to realize the regeneration of the adsorbent (Figure 9C). However, due to the aggregation of MC units, pure SP and poly(SP acrylate) (PSP) suffered considerable photodegradation following the increase in switching cycles (Klajn, 2014). Studies have shown that keeping separate MC units largely inhibits photodegradation and ensures fast and efficient photoisomerization (Radu et al., 2009). Metal–organic frameworks (MOFs) have a high specific surface area, uniform porosity, and flexible skeleton structure (Bachman et al., 2016), which can provide high porosity of adsorbents and good constraints on polymers. Thus, SP/PSP limited within MOFs can effectively isolate MC molecules to improve their stability and performance. This study showed that the light-responsive sunlight-regenerable salt adsorbent (PSP-MIL-53) was a promising energy-saving and sustainable adsorbent for seawater desalination. Under dark conditions, the amphoteric ionic MC rapidly adsorbed cations and anions in water within 30 min. Under sunlight, it isomerized into SP and rapidly released these adsorbed salts within 4 min. It can efficiently capture and release a variety of monovalent and divalent salts prevalent in a large salinity range of natural brine, which has good cycling performance and high ionic adsorption load with the adsorptive capacity of NaCl up to 2.88 mmol g^−1^. The single column seawater desalination experiment showed that the PSP-MOF has a good desalination effect by desalting 2,233 ppm synthetic brackish water to obtain a yield of 139.51 kg^−1^ d^−1^ freshwater and a low energy consumption of 0.11 Wh I^−1^. This sunlight-renewable MOF adsorbent has high cationic and anionic adsorptive capacity, fast adsorption and desorption process, and excellent cycling performance. It provides a novel strategy to directly use renewable solar energy to design functional materials, reduce energy consumption, and improve the sustainability of seawater desalination. Sun-sensitive MOFs can be further functionalized for low-energy, environmentally friendly, and selective mineral extraction (Ou et al., 2020).

**FIGURE 10:**
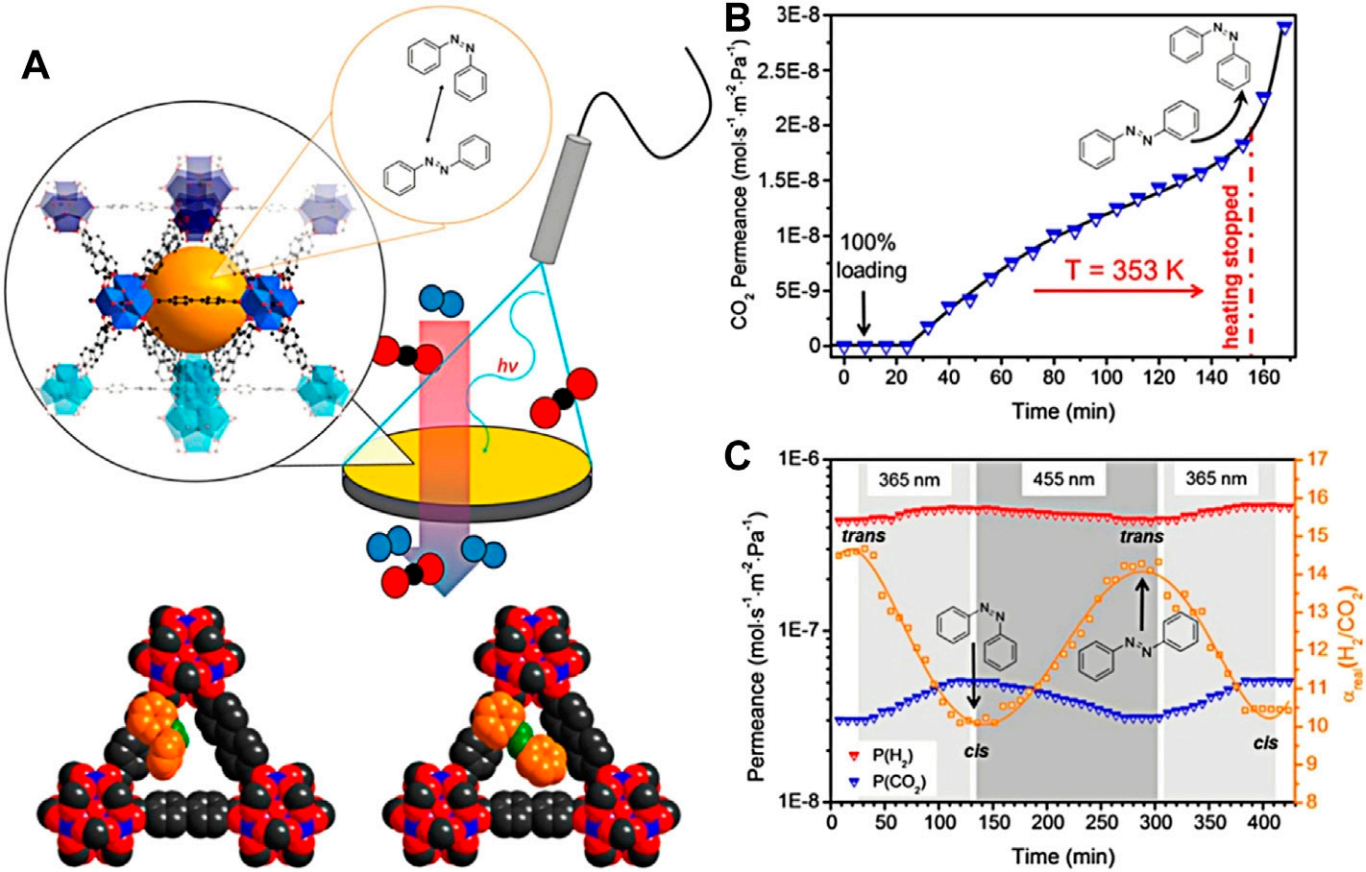
**(A)** Structural changes of Azo in the cavity of UiO-67 under different light conditions, and the schematic diagram of its influence on the transport of gas molecules. The gating mechanism is described by the filling space model. On the left is the aperture window of cis conformation, and on the right is the trans-conformation of UiO-67 and AZB; **(B)** controlled thermal desorption of Azo from the supporting UiO-67 layer. This desorption of Azo (from the membrane fully loaded with Azo) is indirectly traced to an increase in the permeability of CO_2_. During *in situ* desorption process of Azo, the surface of the Azo@UiO-67 film is irradiated by LED at 365 nm continuously, thus transforming Azo from *trans*- to *cis*-isomer. When Azo is desorbed to a certain extent, CO_2_ conduction increases rapidly after heating is stopped; **(C)** reversible gas permeation of the H_2_/CO_2_ mixture during *in situ* reversible switching of UiO-67 under constant and reduced Azo loads. The mixed gas separation factor α (H_2_/CO_2_) changes sinusoidal (Knebel et al., 2017) Copyright @ 2017 (ACS).

The very large pore and internal specific surface area of MOFs make them ideal materials for loading intelligent guest molecules. Jurgen Caro’s group first synthesized a sub-micron thin 200 nm UiO-67 film by the solvothermal method (Knebel et al., 2017). Then, this neat UiO-67 membrane was used as the host material for adsorptive light-responsive guest molecules. The thin layer of the UiO-67 film allows Azo to enter the cavity of UiO-67 as a guest molecule (Figure 10A). By the adsorptive measurements, the authors determined that the pores of UiO-67 were completely filled with Azo and the steric hindrance inhibited any optical switching. After the desorption *in situ* thermal control, Azo can be switched by light with the permeability change of gas to obtain a simple and effective smart material for remote-controlled gas permeability (Figure 10B). The conversion of Azo in the solution and matrix can be observed by UV-vis spectroscopy. Based on the structural changes of Azo, the UiO-67 film with UV/visible light switching performance was constructed in molecular nanopores. The film itself showed the property of gas separation, in which the separate property of the UiO-67 framework was changed by the light-responsive gating effect of the Azo molecule. The reversible separation of H_2_/CO_2_ can be achieved through Azo-loaded UiO-67 films (Figure 10C). Finally, spectroscopic measurements show that Azo guest molecules form π-stacking complexes with MOF macrocyclic hosts, causing light-induced valve opening and closing. A novel supported ultrathin host–guest of the UiO-67 film was successfully synthesized and included with Azo. It is the light stimulus-response, remotely controllable and reversible switching gas separation, which can be applied in the field of sensors or molecular optical switches in microelectronics.

## Conclusion

The design of a photo-responsive artificial nanochannel will contribute to the discovery of information on photo-responsive biological processes in living systems and the development of non-contact regulation of nanofluids with spatial and temporal precision. The photo-responsive host–guest systems have the advantages of reversible assembly and the ability of selective recognition. It is an ideal platform for measuring ionic and molecular transport in atomic precision when it is introduced into the nanochannels (Sun et al., 2020; Qiao et al., 2021). Therefore, the research progress of photo-responsive host–guest-assembled nanochannels in recent years has been reviewed, including the design of nanochannels, regulation of ionic and molecular transport, and application for desalination. The regulation of ionic and molecular transport behaviors can be realized by light stimulation, expanding its application in confined spaces. The different molecular macrocyclic hosts have different properties. For example, CDs as the outside hydrophilic and inside hydrophobic structure can combine with the hydrophobic structure of Azo, which change the wetting of water-based nanochannel surfaces and control the selective transmission behavior of ions and small molecules in an aqueous solution. Furthermore, the selective and controllable transfer of β-CD is realized by specific light stimulation based on host–guest inclusion and de-inclusion. Pillararene has a rigid skeleton structure and hydrophobic cavity, and the upper and lower edges are easy for derivatization and functions. It can form host–guest complexes with photosensitive small molecules of Azo and retinal. Moreover, the upper and lower edges of the functional sites on pillar[6]arene can achieve selective adsorption of different ions and molecules. Therefore, the different types of light-responsive ionic and molecular gating are constructed. It can regulate the selective adsorption, storage, and release of ions and molecules with light response. A MOF has high porosity, large specific surface area, easy to form hydrogen bond network with water molecules, and high conductivity for ions. The photosensitive small molecules are introduced into the nanopore of the MOF to realize the photo-responsive rapid transmission of protons and separation of gas.

Although some progress has been made in the study of light-responsive nanochannels, there are still some challenging tasks in this field. For example, many light-responsive nanochannels are sensitive to UV light. However, UV light is harmful to both samples and operators. Therefore, it is imperative to develop nanochannels which respond to visible and even near-infrared light. In addition, molecular dynamics simulation and COMSOL software should be used to explore the mass transport process in confined nanochannels and further reveal the internal mechanism of ionic/molecular transport behavior under light stimulation. Moreover, the developed photosensitive nanochannels with excellent properties should better integrate the multidisciplinary fields of materials science, biology, and optical physics. Photosensitive nanochannel membranes are also expected to have practical applications in biosensors, biomaterials, and environmental sciences. In conclusion, we hope that this review will provide some insights into the construction of multifunctional artificial nanochannels and contribute to a creative understanding of the principles of light-responsive nanochannels in living systems and their practical applications in our lives.
